# Ontogenetic niche shifts as a driver of seasonal migration

**DOI:** 10.1007/s00442-020-04682-0

**Published:** 2020-06-11

**Authors:** Wimke Fokkema, Henk P. van der Jeugd, Thomas K. Lameris, Adriaan M. Dokter, Barwolt S. Ebbinge, André M. de Roos, Bart A. Nolet, Theunis Piersma, Han Olff

**Affiliations:** 1 Conservation Ecology Group, Groningen Institute for Evolutionary Life Sciences (GELIFES), Univ. of Groningen, Groningen, The Netherlands; 2 Department of Animal Ecology, Netherlands Institute of Ecology (NIOO-KNAW), Wageningen, The Netherlands; 3 Vogeltrekstation, Dutch Centre for Avian Migration and Demography (NIOO-KNAW), Wageningen, The Netherlands; 4 NIOZ Royal Netherlands Institute for Sea Research, Department of Coastal Systems, and Utrecht University, Den Burg, Texel, The Netherlands; 5 Cornell Lab of Ornithology, Cornell University, 159 Sapsucker Woods Road, Ithaca, NY 14850 USA; 6 Wageningen Environmental Research, Wageningen Univ. and Research, Wageningen, The Netherlands; 7 Department of Theoretical and Computational Ecology, Institute for Biodiversity and Ecosystem Dynamics (IBED), Univ. of Amsterdam, Amsterdam, The Netherlands

**Keywords:** Barnacle goose, Dark-bellied brent goose, Humpback whale, Matrix population modelling, Ontogeny, Pacific salmon, Reproduction, Seasonal migration

## Abstract

Ontogenetic niche shifts have helped to understand population dynamics. Here we show that ontogenetic niche shifts also offer an explanation, complementary to traditional concepts, as to why certain species show seasonal migration. We describe how demographic processes (survival, reproduction and migration) and associated ecological requirements of species may change with ontogenetic stage (juvenile, adult) and across the migratory range (breeding, non-breeding). We apply this concept to widely different species (dark-bellied brent geese (*Branta b. bernicla*), humpback whales (*Megaptera novaeangliae*) and migratory Pacific salmon (*Oncorhynchus gorbuscha*) to check the generality of this hypothesis. Consistent with the idea that ontogenetic niche shifts are an important driver of seasonal migration, we find that growth and survival of juvenile life stages profit most from ecological conditions that are specific to breeding areas. We suggest that matrix population modelling techniques are promising to detect the importance of the ontogenetic niche shifts in maintaining migratory strategies. As a proof of concept, we applied a first analysis to resident, partial migratory and fully migratory populations of barnacle geese (*Branta leucopsis*). We argue that recognition of the costs and benefits of migration, and how these vary with life stages, is important to understand and conserve migration under global environmental change.

**Electronic supplementary material:**

The online version of this article (10.1007/s00442-020-04682-0) contains supplementary material, which is available to authorized users.

## Introduction

Because foraging abilities and vulnerability to predation risk tend to vary with body size, many species change their food and habitat use in the course of their life. Such changes have been termed ontogenetic niche shifts (ONS) (Werner and Gilliam 1984). As species undergo ONS, the successive stages of life will be characterized by different ‘ecologies’ and thus a different relative importance of various limiting environmental conditions (de Roos and Persson 2013). ONS are especially well-known in species with complex life cycles such as insects and amphibians (Werner and Gilliam 1984). Among animals with less complex life cycles, ONS are less obvious but often occur as well, especially in fish and reptiles, if only for the simple reason that young are smaller than adults, and body size correlates with predation risk (Sinclair et al. 2003; McLeay et al. 2009), dietary range (Matich et al. 2019), stress resistance (Xu and Ji 2006) and many other physiological and ecological constraints (Peters 1983). Yet even in birds and mammals ONS may occur when specific adaptive traits need time to develop, for example salt glands (Hannam et al. 2003; Gutiérrez 2014) or muscular gizzards to crush hard food items (Hannam et al. 2003; van Gils et al. 2003; van den Hout et al. 2014).

Another widespread biological phenomenon is migration, the persistent movement with directional bias, usually over larger spatial scales (Fryxell et al. 2011). The main factors that control demographic processes, i.e. survival and reproduction, are likely to vary with season and along the migratory trajectory (Piersma and Baker 2000; Taylor and Norris 2010). However, species undergoing ONS do not always migrate (Miller and Rudolf 2011; de Roos and Persson 2013; Rudolf and Rasmussen 2013; Preston et al. 2014; Sanchez-Hernandez et al. 2019) and migratory species do not all undergo ONS, for example cranes that despite some age-related size differences are exposed to the same predators, have basically the same diet and migrate together (Teitelbaum et al. 2016). Nevertheless, a move to environments which are suitable for specific life stages—such as immature stages—has been considered an ultimate reason for migration (Rasmussen et al. 2007; Fryxell et al. 2011). So far, the generality of this idea has been little explored.

## Migration and ontogenetic niche shifts

For an understanding of migration, it is an important question whether migratory decisions can be understood as driven by the ecological requirements of the adults alone, or whether the ecological constraints and requirements of immature stages (that is ONS) play a role as well. Migration has independently evolved multiple times in birds (Piersma et al. 2005), mammals (Avgar et al. 2014), fish (Goss et al. 1988; Roff 1988) and invertebrates (Roff and Fairbairn 2007). Most authors have considered seasonal migration primarily an adaptation for exploiting seasonal peaks in resource availability (Alerstam et al. 2003; Newton 2008; Dingle 2014). An alternative driver of migration which has recently been put forward, is the aim of organisms to maintain site fidelity to familiar productive breeding locations, with seasonality forcing a non-breeding departure from these locations (Winger et al. 2019). Modelling studies have suggested that seasonal migration rather than residency should be the rule rather than the exception, as long as at least two different habitats are available and accessible, which are associated with seasonal differences in fitness gains, and there exists density-dependent regulation (Holt and Fryxell 2011; Fryxell and Holt 2013; Somveille et al. 2018). However, migration comes with costs, including the energetic costs (Drent and Piersma 1990), possibly increased mortality risk (Klaassen et al. 2014), information costs (Lok et al. 2015), as well as costs of adjusting body composition or immune defense to cope with particular conditions (Buehler and Piersma 2008; Buehler et al. 2010). When the costs exceed the benefits, migration is not an evolutionary stable strategy (Fryxell and Holt 2013; Avgar et al. 2014). However, fitness costs and benefits may differ between life-stages (de Roos and Persson 2013). Therefore, as an extension to the existing migration theory framework, we here explore the importance of ONS in the evolution and maintenance of seasonal migration. We attempt to parameterize the costs and benefits of migration by quantification of life stage-specific demographic processes.

Successful juvenile survival may require different environments than what is best for the survival of the reproductively active adults. As emphasized by the common finding that young birds can remain in the non-breeding environment for up to several years (van Dijk et al. 1990; McNeil et al. 1994; Summers et al. 1995), many avian migrants could potentially stay and survive year-round in their wintering grounds. Yet, as adults they undertake annual migrations to specific areas for their reproduction, which in some cases may decrease their own probability of survival but are a necessity for successfully producing offspring (Klaassen et al. 2014; Loonstra et al. 2019) (but see Leyrer et al. 2013; Conklin et al. 2017). The differences between environmental suitability for growing chicks and adults may also give rise to “conflicts” between the ecological and physiological requirements within a population of individuals at different life stages. This is shown by Arctic-breeding geese, for example, which often undertake long moult migrations once released from parental care due to nest failure or loss of dependent offspring, while successful parents are forced to moult on the breeding grounds (Reed et al. 2003). Conflicts between the optimal habitat for adults and juveniles can be overcome in different ways. Adults can, via extensive parental care, create a suitable environment for their young, as do altricial birds which actively feed their young to overcome the problem of lack of mobility and food catching capacity of their chicks (Starck and Ricklefs 1998). A disadvantage of this intense care-taking is the energetic cost involved, which may negatively affect adult survival and future reproduction (Daan et al. 1996). Another solution to provide offspring with a suitable environment is for the reproducing adults to move to habitats especially suitable for growing young.

Many insect and amphibian species have found intriguing solutions to the clear conflict between the ecological requirements of the terrestrial adults and the aquatic larvae. The conflicts between juvenile and adult requirements in these taxa are generally solved through metamorphosis (Brink et al. 2019). Since larvae are not provided with parental care, juveniles and adults have evolved to live in completely different habitats, which can occur next to each other on small spatial scales (for example, a pond and its surrounding marsh vegetation) (Knight et al. 2005). However, for many vertebrates it is not possible for parents and young to occupy different ecosystems, since juveniles need an initial phase of parental nutrition and protection. In these cases, adults have to move towards an ecosystem which provides suitable physiological and ecological conditions for their young. Such habitats can be typically seasonal with peaked resource availability, often proposed as a main driver of migration, but also with suitable food, benign climatic conditions and relatively few predators for juveniles. Sometimes, these habitats can be found close-by. Eurasian curlews (*Numenius arquata*) in the UK use grasslands during reproduction, whereas outside the breeding season they use nearby mudflats as the main habitat (Durell 2000). However, when the best habitats for reproduction are far away, or have become spatially separated over evolutionary timescales, long-distance migration may evolve as a strategy that enables successful reproduction (Winkler et al. 2016). We propose that ONS can be an important explanation for the evolution and maintenance of such seasonal migrations between distant breeding and nonbreeding ranges.

In this paper, we present a scheme that encompasses the full annual cycle of migrants for different ontogenetic stages, such that the consequences of external factors acting differently on different ontogenetic stages can be adequately understood. We illustrate the usefulness of this scheme with a number of well-studied species, including birds, fish and mammals. On this basis we aim to show that the assignment of costs and benefits of migration to life stage components help us establish the presence and spatial location of external bottlenecks, like nutritional problems and risks (Buehler and Piersma 2008), and hence help predict population change under novel environmental conditions.

## A scheme to integrate age- and season-specific demographic rates

**Fig. 1:**
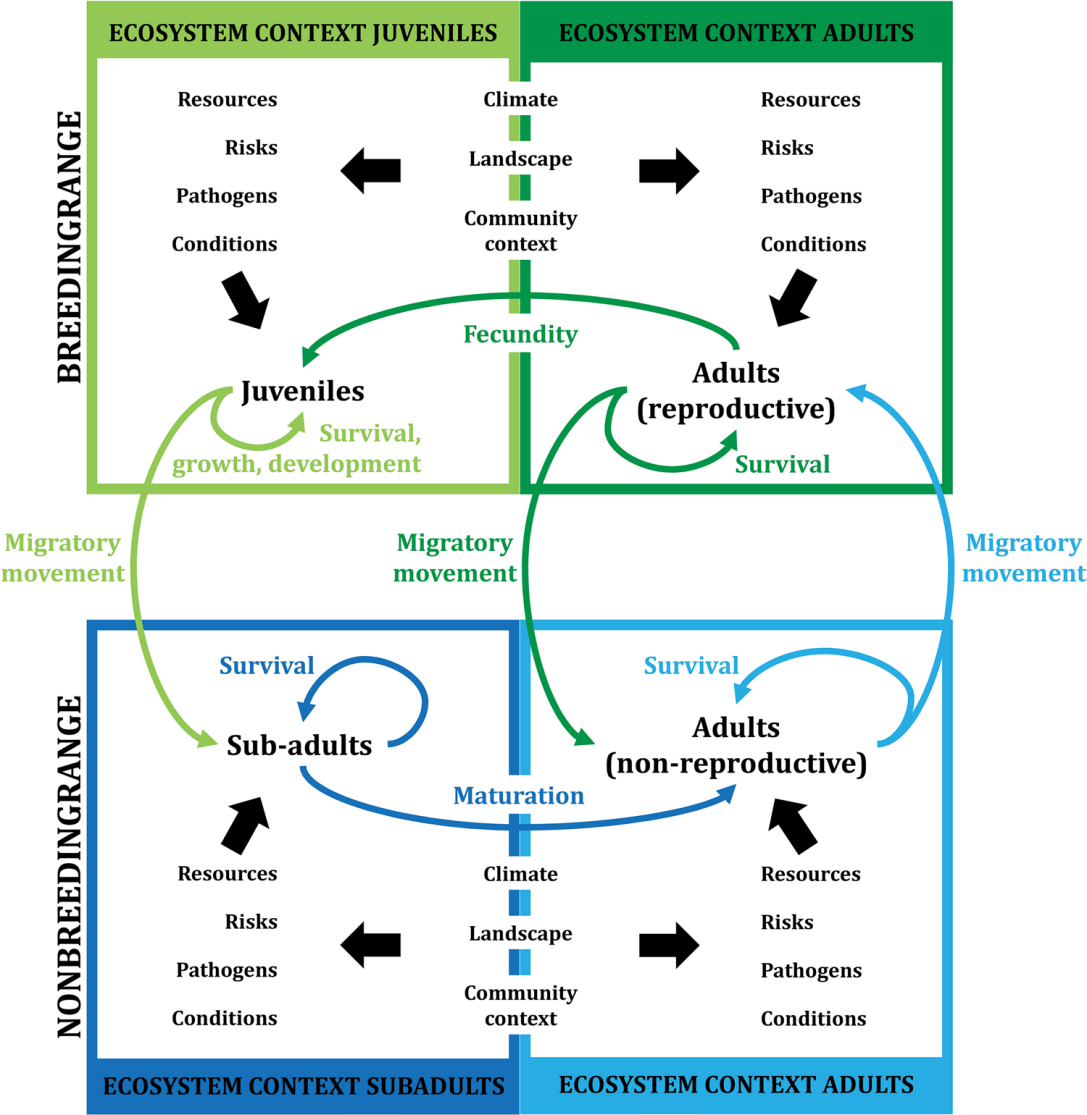
Scheme representing the life cycle of a migratory species. The inner circle shows the life stages (in black), the main life history processes (in color), in both the breeding and non-breeding range and the transition between the different life stages (colored arrows). The boxes represent the ecosystem contexts of the different life stages. These ecosystem contexts influences the life history processes through factors (connected to the life history stages with black arrows). The ecosystem context, and the factors within those, will differ for the different life stages (for instance, juveniles in the breeding range will perceive a different ecosystem context than the adults). The colour of the arrows and of the life history processes next to those indicates which ecosystem context is relevant for that life history process

As a starting point we use the two-stage life cycle model proposed by de Roos and Persson (2013). We assume that a migratory range can be simplified into a breeding and a non-breeding habitat (Fig. 1). Note that for simplicity the phase of long-distance movement itself, i.e. when animals are en route and exposed to the vagaries of weather, currents and wind (Shamoun-Baranes et al. 2010; Gill et al. 2014), is taken out of the equation. The breeding and non-breeding habitats differ with respect to the external factors affecting vital rates. Thus, the adult-specific ecosystem context (assembly of external factors affecting a particular life stage) of the breeding range affects adult fecundity and survival, whereas the juvenile-specific ecosystem context affects the survival, growth and development of juveniles before they migrate to the non-breeding range. Following migration, life stage-specific ecosystem contexts of the non-breeding range determine adult survival and further development of their young as sub-adults until they eventually mature into adults (Fig. 1). Often, sub-adults take more than a year to mature and survive in the non-breeding range before first migrating to the breeding grounds (Summers et al. 1995; Hockey et al. 1998).

The time needed to complete a full life cycle differs strongly between taxa. Seasonal migrants like many migrant amphibian, birds and mammal species, typically take one year to complete a full cycle of visiting the non-breeding and breeding range, whereas this may take many years for fish and reptile species with longer and sometimes unrepeated life cycles (Hedenström 2006; Southwood and Avens 2010; May 2013; Avgar et al. 2014; Sinsch 2014). Many migrating insect species complete a full migration cycle in several generations (Altizer et al. 2011; May 2013; Brattström et al. 2018). Nonetheless, the proposed scheme has enough generality to be applicable to a wide range of both seasonal migrants, which occupy different habitats during different seasons, and life cycle migrants, which utilize different habitats during different life stages.

## Quantifying population dynamics of migrants with ontogenetic niche shifts

We suggest that the explicit consideration of ONS in demographic analyses is vital for understanding migratory systems. For this, the scheme presented in Fig. 1 needs to be translated into demographic models. Demographic data can then be used in matrix population models, which are a well-developed tool in analyzing population dynamics and can be used to detect what demographic processes are limiting population growth (Caswell 2001; Caswell et al. 2018). Matrix models have been used to study migratory species (Sillett and Holmes 2005; Dinsmore et al. 2010; Flockhart et al. 2015). In most cases however, breeding and non-breeding seasons are not considered separately, even though vital rates, like survival, may differ strongly between these seasons (but see Rushing et al. 2017), and a better approximation of what the limiting demographic processes as well as  the causing factors are, can be achieved by considering time steps which are smaller than one year (Rakhimberdiev et al. 2015; Piersma et al. 2016).

**Fig. 2:**
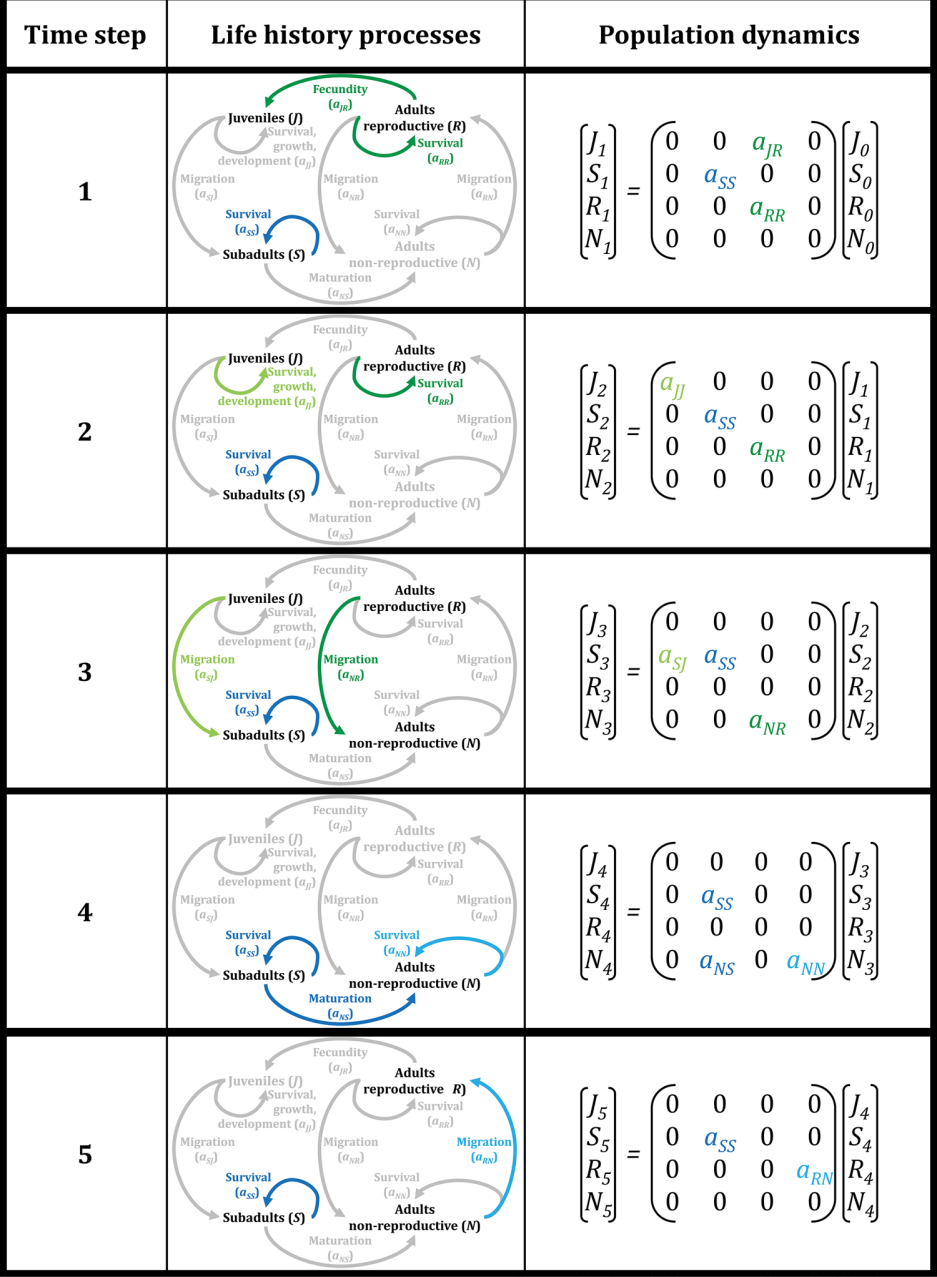
Outline of how our conceptual framework can be captured in a matrix population model. The model has five time steps to describe a whole migratory life cycle. The second column shows in color the part of the life cycle which is associated with the time step indicated in the first column. The vital rates are noted like *a*_yx_, which indicated the rate with which individuals transition from stage x to stage y, or in case of reproduction, the contribution of stage x to stage y. The “non-active” parts of the life cycle are presented in grey. The third column shows the associated matrix formulation to calculate numbers of juveniles (J), subadults (S), reproductive adults (R) and non-reproductive adults (N) at any time step for a migratory population

A general model comprises four life stages (adult reproductive, adult non-reproductive, juveniles and sub-adults: see Fig. 2). A full cycle consists of five time steps, which can each be characterized by different matrices with vital rates (Fig. 2). The first time step is the early breeding season, when juveniles are produced. The second step represents the phase in which juveniles are growing and developing. The third time step involves migration towards the non-breeding grounds. The fourth step comprises the non-breeding season. The final stage involves migration of adults towards the breeding grounds (Fig. 2).

## Empirical examples

The migratory systems of the full migrants dark-bellied brent goose (*Branta bernicla bernicla*), humpback whale (*Megaptera novaeangliae*) and Pacific salmon (*Oncorhynchus* sp.) are all well-studied and characterized by the large differences between the breeding and non-breeding range. The examples come from the worlds of birds, mammals and fish and differ not only in their mode of movement (flying and swimming), but also in their type of migration and degree of parental care. Whereas brent goose and humpback whale migrate seasonally and show parental care, Pacific salmon only return to their breeding range once without exhibiting parental care. To show the generality of the proposed scheme, we will now apply it to these three distinct taxa and map which costs (factors which negatively affect vital rates) and benefits (factors which positively affect vital rates) are associated with the different life stages during the different seasons (Table 1).

**Table 1:** Summary of the benefits (B) and costs (C) for brent goose, humpback whale and pacific salmon of the different life stages on different spatial locations

Life stage	Spatial location	Brent goose	Humpback whale	Pacific salmon
Juvenile	Breeding range	Arctic tundra B: high quality, non-saline plants (developing salt glands), long days, low pathogen load C: high predation by e.g. Arctic fox	Equatorial waters B: warm waters (development of thermoregulatory system), less predation by killer whale	Freshwater streams B: non-saline conditions (developing salt tolerance), invertebrate availability C: predation by fish (e.g. trout), avian predators (e.g. kingfisher) and mammals (e.g. otter)
Sub-adult	Non-breeding range	European estuaries B: mild winter, family support C: saline conditions, food depletion	Arctic waters B: high food availability C: more predation by killer whale, cold waters	Oceans B: krill, invertebrates C: predation by avian predators (e.g. cormorant), fish (e.g. shark) and marine mammals (e.g. seal), pathogens (e.g. sea lice), fishing
Adult	Breeding range	Arctic tundra B: high quality food, long days C: too early arrival: no food available yet	Equatorial waters C: no food available	Freshwater streams C: predation by e.g. bears
	Non-breeding range	European estuaries B: mild winter C: seagrass decline	Arctic waters B: high food availability	Oceans B: fish, krill C: predation by avian predators, fish and marine mammals, pathogens, fishing

### Dark-bellied brent goose

This herbivorous bird has its breeding range on the tundra in Siberia, whereas its non-breeding range consists of coastal temperate Europe (Ebbinge et al. 1999; Ganter 2000; Green et al. 2002). The adults arrive on the breeding grounds when these are still frozen, covered in snow and without accessible food (Ebbinge and Spaans 1995) and incur substantial risk and cost to increase the survival chances of their young, which especially benefit strongly from the non-saline conditions, because of undeveloped salt glands (Stolley et al. 1999), the high quality food (Richman et al. 2015), the long days (Eichhorn et al. 2019) and possibly the low pathogen load of the breeding range (Buehler et al. 2009) (Fig. S1). Only later in the breeding season adults may benefit from the Arctic conditions, especially during their annual wing-moult, when they are temporarily flightless (Ebbinge et al. 1999). Costs affecting both adults and young in the breeding range include the high predation rates by e.g. Arctic foxes, Taimyr gulls and snowy owls, even though the exact predation pressure depends on the lemming cycle (Summers 1986; Ebbinge and Spaans 2002; de Fouw et al. 2016). In its coastal temperate non-breeding range, brent geese utilize different habitats, ranging from seagrass beds to lower salt marshes to coastal agricultural fields (Dokter et al. 2018). Especially adult brent geese, with active salt glands, profit from the saline conditions, which provide high-quality salt marsh plants, seagrasses (*Zostera* spp.) and seaweeds (*Ulva* spp.) (Ponsero et al. 2009; Fokkema et al. 2016), little competition with other herbivores (Fox 1996; Percival and Evans 1997), and potentially few parasites and pathogens (Piersma 1997; Figuerola 1999).

### Humpback whale

**Fig. 3:**
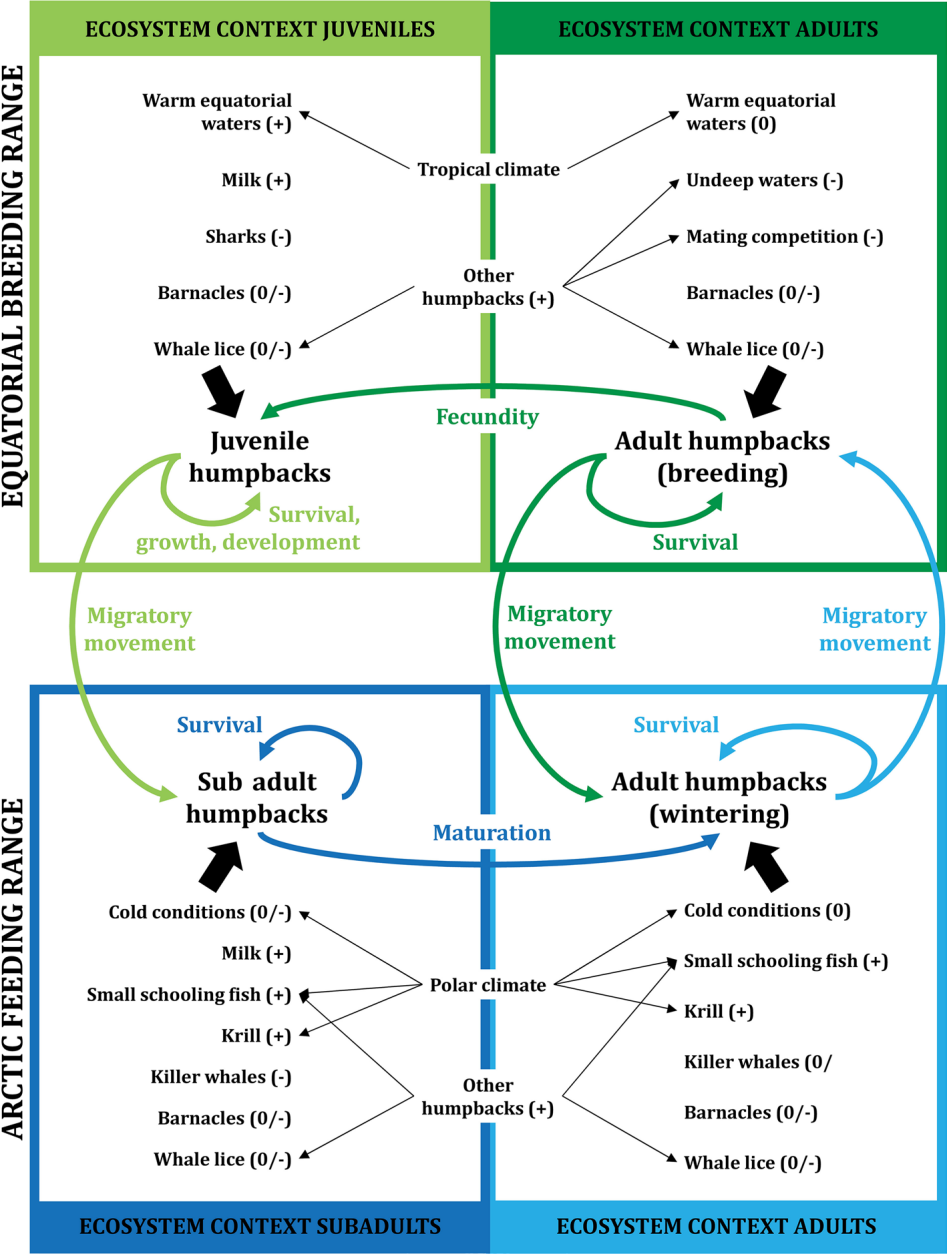
Example life cycle of a marine mammal, the humpback whale. Humpback whales are a clear example of a species in which migration to the equatorial breeding range is mainly beneficial for the juveniles, which cannot cope with the cold conditions of the arctic feeding range. Adults do not profit from migration in terms of resources, since those are largely lacking in the warmer equatorial waters

Humpback whales breed in warm equatorial waters and spend their summer in its non-breeding range, consisting of the Arctic oceans (Clapham 1996). The juveniles profit from the warm conditions in the breeding range, which reduce thermoregulatory costs, and from low predation pressure by killer whales, while feeding from the fat reserves of their parents (Fig. 3). Contrastingly, the adults strongly deplete their energetic reserves in the breeding range, and hence pay energetic costs: there is no food available, and thus the adults rely solely on their fat reserves (Clapham 1996), making them a typical example of a ‘capital breeder’ (Drent and Daan 1980). Furthermore, intense mating competition forces the females to shallow waters, involving the risk of stranding (Craig et al. 2014).

During the non-breeding season in the rich Arctic oceans, humpbacks can build large fat stores, since large amounts of fish and krill are available (Ryan et al. 2014). However, especially for the sub-adults, the non-breeding range comes with costs including predation pressure by killer whales (McCordic et al. 2014) and relatively cold conditions, which require a thick fat layer to survive.

### Pacific salmon

Pacific salmon complete one migratory cycle in their lifetime. After hatching in their freshwater breeding range, they subsequently migrate to the marine non-breeding range and eventually return after several years as adults to the breeding range, where they die after spawning (Schindler et al. 2003; Altizer et al. 2011; Keefer and Caudill 2014; Schindler 2019) (Fig. S2). Apart from the salmon being a keystone species for the ecosystems of freshwater streams and the surrounding terrestrial systems (Willson and Halupka 1995; Helfield and Naiman 2006; Subalusky and Post 2019), the conditions provided by the freshwater breeding grounds are essential to the juveniles. The fact that they are still developing their salt tolerance has been suggested as the main reason for this (McCormick 1994). Costs in the breeding range include high predation pressure by fish, avian and mammalian predators (Metcalfe et al. 1999).

In the non-breeding range, sub-adults first remain in brackish estuaries, where they further develop into adult salmon (MacFarlane and Norton 2002; Hanson et al. 2013). Adults utilize the open oceans, where they benefit from the presence of food sources, as krill, fish and squid (Keeley and Grant 2001; Bargu et al. 2002; Aydin et al. 2005). Costs include predation by avian predators, fish species and marine mammals (Tasker et al. 2000; Hauser et al. 2008; Williams et al. 2011; Carlisle et al. 2015).

The evolutionary origin of a migratory species can give insight in the processes driving the migration patterns. Whether the evolutionary origin of salmon and salmonids in general lies in the freshwater or marine habitat has been highly discussed, but most evidence now points towards a freshwater origin (Alexandrou et al. 2013; Zhivotovsky 2015). Within species of salmonids, like the brown trout (*Salmo trutta*), different strategies can coexist, with populations at higher latitudes more often migrating to marine environments, while this is less common at lower latitudes (McDowall 1997). At lower latitudes, freshwater rivers and lakes provide a food-rich environment, whereas tropical oceans are poor. On the other hand, at higher latitudes the marine environment is extremely rich. For adult Atlantic salmon (*Salmo salar*) evolving towards the use of oceans, instead of freshwater rivers and lakes is likely to have been beneficial from a food perspective (McDowall 1997). However, returning to freshwater environment for spawning is necessary, since the larvae have not evolved towards immediate tolerance to the saline environment (McCormick 1994), suggesting that ONS play an important role as driver of the migration patterns in salmon.

### A partial migrant: different strategies of the barnacle goose

The species we have considered in the examples above are all full long-distance migrants. However, the role of ONS in explaining migration can be further explored by comparing different strategies in partially migratory populations, which are composed of a mixture of resident and migratory individuals (Chapman et al. 2011). As a first step and a proof of concept (Fig. 2), we applied a preliminary analysis to detect the most successful current migration strategy and to establish the effect of using multiple habitats on different life stages of the barnacle goose. We chose this species since demographic data are available for three subpopulations with different migration strategies and because its ecology shows similarities to that of the brent goose, which we have presented as an example earlier, but lacks the non-migratory and short-distance migrant strategies and is still fully migratory.

Currently, the barnacle goose population wintering in The Netherlands and Germany consists of three subpopulations with different breeding strategies: the first, ancestral strategy, which was used by the entire population before the 1970s, involves long-distance migration to reach breeding grounds in the Russian Arctic (mainly Novaya Zemlya and Vaigach) (Larsson and Forslund 1994; van der Jeugd et al. 2009). The second strategy has emerged during rapid growth of the barnacle goose population in the 1970s and onwards and involves a much shorter migration and breeding at a former stop-over site in the Baltic (Larsson and Forslund 1994; van der Jeugd et al. 2009). Finally, the most recent strategy emerged in the 1980s and involves complete residency with birds breeding in their Dutch wintering grounds (van der Jeugd and Kwak 2017). Following our hypothesis, we would expect that the ecosystem context provided by high latitudes is crucial in maintaining the long-distance migratory strategy.

**Fig. 4:**
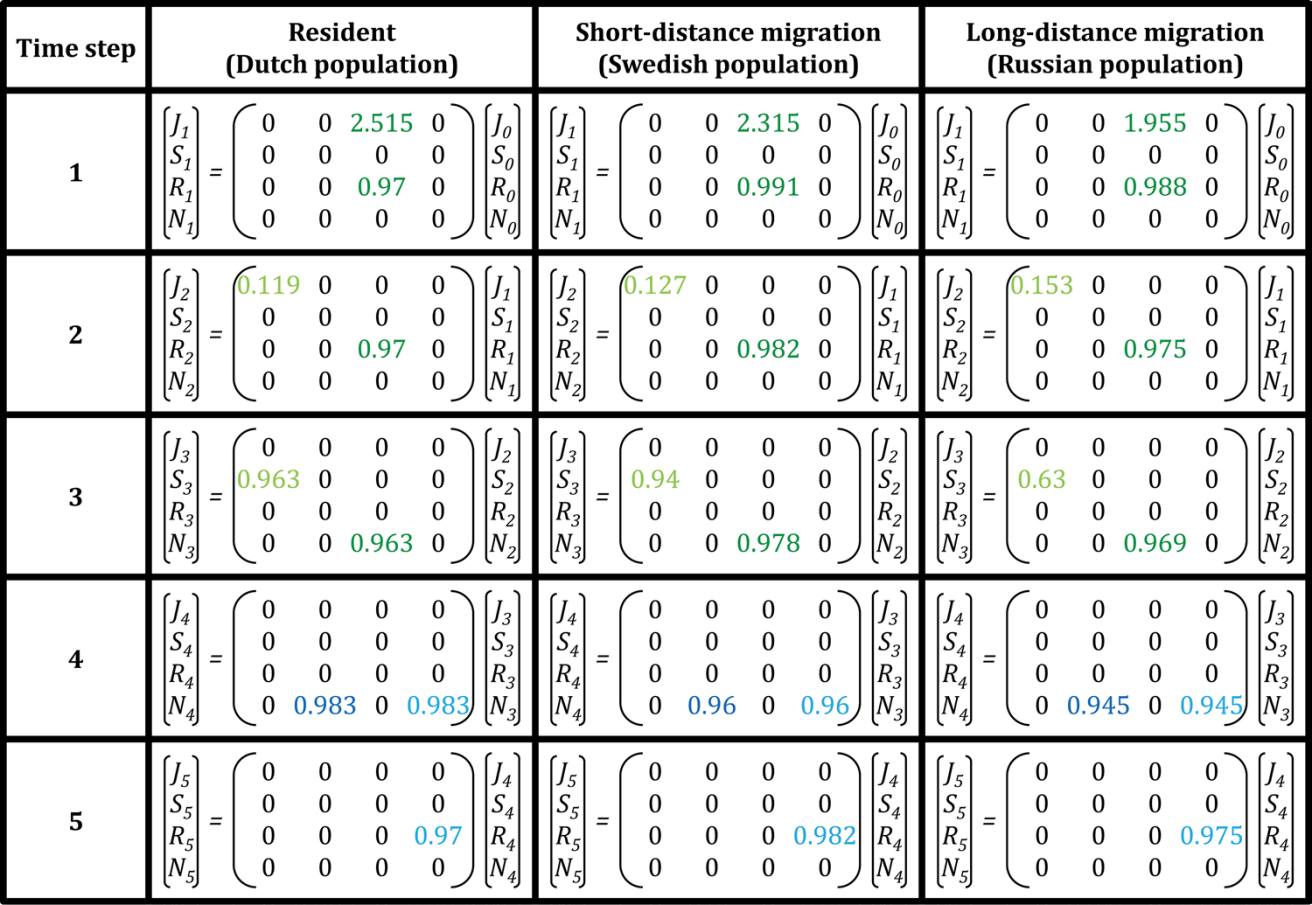
Matrices for three different migration strategies in the barnacle goose. Data is obtained from literature (see supplementary methods and results). Lambda, the population growth rate, of the resident population is 1.139, that of the short distance migrating population is 1.157 and the Lambda of the long-distance migrating population is 1.034. These differences are caused by the differences in partial vital rates as can be seen from the values in the matrices

We used existing publications (Larsson and Forslund 1994; van der Jeugd and Larsson 1998; van der Jeugd et al. 2009; van der Jeugd 2013) in combination with some unpublished data to obtain the vital rates for the different subpopulations (see Supplement for details). Because the studies we used to establish the vital rates were not set up to fit our model, information on certain vital rates, for example survival, was not available for all the time steps separately. Despite such issues, our analysis did reveal that overall the Russian subpopulation is growing slowly (*λ* = 1.034 during 2003–2014), whereas the short-distance migrants that migrate to the Baltic and the non-migratory population which remains in The Netherlands were growing rapidly during the periods they were studied (*λ* = 1.157 for the Baltic subpopulation in 1984–2001 and *λ* = 1.139 for the Dutch subpopulation in 2004–2012). The differences in the population growth are caused by differences in the partial vital rates and match population growth rates based on counts in the referred periods remarkably well. Meanwhile, the growth rate of the relatively recently established short-distance migrant population has declined, and we also expect the growth rate of the resident population to decline and approach 1 because of density-dependence on the breeding grounds. However, despite a currently lower population growth rate, the long-distance migratory strategy was characterized by a higher survival of chicks during the pre-fledging phase, offset by lower survival during later stages (Fig. 4). The population growth rate of the long-distance migrants was also much less sensitive to changes in juvenile survival (*a*_jj_) than the resident and short-distance migrants (Table S1). This fits our expectation that juveniles benefit most from the conditions in the high-latitudinal breeding range and also is in line with the green wave hypothesis, which predicts higher quality food for birds which migrate northwards along with the early spring quality peak of the vegetation (van der Graaf et al. 2006; Kolzsch et al. 2015). Based on our analysis, the different populations are all viable, albeit at different rates, suggesting that the long-distance migratory strategy is so because of the advantages of the juveniles in the Arctic.

## Discussion

The suggestion that migration may serve “to find suitable habitats for different life stages with markedly different physical needs and ecological constraints” has been put forward before (Fryxell et al. 2011), but without mentioning ONS specifically. Here we propose a scheme to evaluate the importance of ONS for our understanding of migration. The above empirical examples show the generality that conditions in the breeding range are beneficial for juveniles rather than for adults, in terms of abiotic conditions as found in humpback whales and salmons, or because of better quality food or higher food availability, for barnacle and brent geese. In brent geese, adults using more marine resources have a slower decline in body condition in winter than adults feeding more on terrestrial resources (Inger et al. 2010), and hence seem to profit from the saline conditions in their non-breeding range. In humpback whales, the adults can only feed in the non-breeding range and in salmon adults also profit from the food availability provided by the marine environment. These commonalities confirm our hypothesis that ONS play a considerable role in the maintenance and evolution of migration and that migratory habitat decisions should therefore be viewed as a trade-off between what is suitable for juveniles and adults. This complements the long-standing idea of seasonal migration being mostly driven by organisms tracking resource peaks (Drent et al. 2003; van der Graaf et al. 2006). Differences between life stages affect the spatial configuration of the fitness landscape and eventually cause species to migrate over long distances. Furthermore, understanding the costs and benefits associated with different life stages will be critical for understanding current migration systems and their development under global change.

Parameterization of the matrix population model requires detailed data, which may not be fully available or complete for populations of interest. If data are not available for all time steps, larger steps could in theory be made. For example, rather than calculating fecundity (*a*_jr_) and juvenile survival on the breeding grounds (*a*_jj_) separately, a combined measure such as the number of fledglings could be used. However, in order to analyze the relative benefits of migration and ONS, it is essential to separate a measure for reproduction on the breeding grounds from survival during the first migration and winter period.

Our general scheme can easily be extended to include other important factors which affect the demographic processes in migratory species. For example, we admit the importance of seasonal interactions and carry-over effects, which emerge when, for example, the non-breeding conditions may strongly influence the reproductive outcome during the next breeding season, as has been described for various migratory species (Norris and Marra 2007; Harrison et al. 2011; Senner et al. 2015). In such cases, a demographic process in the general scheme is partly explained by the ecosystem context of the current habitat and partly by that of the previous habitat during the previous season. Furthermore, the conditions on staging sites along the migration route have been shown to play a large role in the demography of migratory species (Baker et al. 2004; Rakhimberdiev et al. 2018). Including such staging sites is another possible extension of the general scheme.

Like other migratory species, the species in the empirical examples are increasingly affected by global change (Chaparro-Pedraza and de Roos 2019). In brent geese traditional top reproductive years have become scarce; these were associated with peak lemming abundance in the tundra, resulting in breeding seasons with low predation pressure on birds, but lemming cycles are faltering because of less favourable snow conditions (Kausrud et al. 2008; Gilg et al. 2009; Nolet et al. 2013). In barnacle geese, a reduction in gosling survival is found due to an increase in phenological mismatches between the moment of peak food availability and hatch in earlier springs (Lameris et al. 2018). In salmon, higher temperatures result in faster growth and development, but lower survival and reproduction when temperatures increase too much (Crozier et al. 2008). For humpback whales, higher temperatures will cause an even lower food availability in the equatorial waters due to increased extinction rates (Jones and Cheung 2015).

In order to make predictions about migratory system of a species, the different strategies that a species is currently using or could potentially use should be compared, like we did for barnacle geese. The non-migratory strategy in that example lacks the migration steps and as a consequence, survival is higher during this time step. The question is whether a species is doing equally well (or better) without the burden of migration but thereby losing the advantages of using different habitats that are spatially and ecologically separated. The explicit partitioning of vital rates into separate components for age-classes and ecosystem contexts, as we propose, and accurate parameterization of the accompanying matrix population models, may help to clarify how costs and benefits in different ecosystem contexts collectively drive migratory decisions.

## Electronic supplementary material

Below is the link to the electronic supplementary material.

Supplementary file1 (PDF 1374 kb)
