# Supplementary material for: Ontogenetic niche shifts as a driver of seasonal migration
Source: Oecologia. 2020 Jun 11;193(2):285–97. doi: 10.1007/s00442-020-04682-0 (PMC7320946; doi:10.1007/s00442-020-04682-0)
Supplement: Supplementary file 1 — Supplementary file1 (PDF 1374 kb) [file 442_2020_4682_MOESM1_ESM.pdf]

SUPPLEMENT

Ontogenetic niche shifts as a driver of seasonal migration

Wimke Fokkema[1] • Henk P. van der Jeugd[2,3] • Thomas K. Lameris[2,4] • Adriaan M. Dokter[2,5] • Barwolt S. Ebbinge[6] • André M. de Roos[7] • Bart A. Nolet[2,7] • Theunis Piersma[1,4] • Han Olff[1]

Corresponding author: B.A Nolet, (https://orcid.org/0000-0002-7437-4879) ✉ (b.nolet@nioo.knaw.nl)

[1] Conservation Ecology Group, Groningen Institute for Evolutionary Life Sciences (GELIFES), Univ. of Groningen, Groningen, The Netherlands

[2] Department of Animal Ecology, Netherlands Institute of Ecology (NIOO-KNAW), Wageningen, The Netherlands

[3] Vogeltrekstation, Dutch Centre for Avian Migration and Demography (NIOO-KNAW), Wageningen, the Netherlands

[4] NIOZ Royal Netherlands Institute for Sea Research, Department of Coastal Systems and Utrecht Univ., Den Burg, Texel, the Netherlands

[5] Cornell Lab of Ornithology, Cornell University, 159 Sapsucker Woods Road, Ithaca, NY 14850, USA

[6] Wageningen Environmental Research, Wageningen Univ. & Research, Wageningen

[7] Department of Theoretical and Computational Ecology, Institute for Biodiversity and Ecosystem Dynamics (IBED), Univ. of Amsterdam, Amsterdam, the Netherlands

Defining the different time steps and life stages

In order to be able to compare the different populations of the barnacle goose, we divided the year into the different time steps recognized in the model (Fig. S1). Time step 1 (the beginning of the breeding period, in which chicks are produced) ranged from the 15th of May to the 15th of June. Time step 2 (the remaining breeding period until migration to the wintering range) ranged from the 15th of June to the 15th of August. Time step 3 (migration towards the wintering range) was estimated from 15th of August to the 31st of October. Time step 4 (wintering) ranged from the 31st of October to the 15th of March. Finally, time step 5 (migration towards the breeding grounds) ranged from 15th of March to the 15th of May. Note that this division of periods in solely for comparison between the different strategies. The absolute time ranges are likely to differ between the different strategies. The long-distance migrants have to migrate further than the short-distance migrants, making migration periods for this population longer. This is not taken into account here.

The different life stages were distinguished according to the model. The young produced during time step 1 were the number of eggs laid by an individual female. The period in time step 2 ranged from eggs to the point of migration (including a post-fledging period). One month of this period was considered survival until fledging. The other month was considered to be post-fledging survival. Subadults were considered as the chicks which were still alive after migration. Even though barnacle geese do not breed until they are 2 of 3 years old, we did not consider the subadults which were left at the wintering grounds. Firstly, since those subadults migrate with the adults to the breeding range and secondly because data on this group of birds is not widely available.

Data used and calculation of vital rates

Parameters in the model were established using vital rates from different sources of literature. For the Netherlands, data originated from van der Jeugd (2013). These data were collected over a period from 2004 to 2012. For this population yearly survival was available (0.88). However, there was no separate data for the survival of different life stages or periods of the year. We did distinguish between a hunting and non-hunting period. Since 2005-2006 this population is hunted during the summer months (time steps 1, 2, 3 and 5). During the winter months hunting is prohibited. Since we have data available from years in which the geese were not hunted (2004-2006) and from the years in which the geese were hunted (2006 onwards), we have two different annual survival rates: with hunting the annual survival is 0.8346. Without hunting the annual survival is much higher (0.9543). We used the annual survival with hunting to calculate survival during the time steps 1, 2, 3, and 5, as follows: $0.8346^{x/12}$, where x is the number of months associated with the different time steps (for time step 1: x=1, for time step 2: x=2, for time step 3: x=2.5, for time step 5: x=2). This gave the following survival rates for the different periods: 1: 0.985, 2: 0.970, 3: 0.963, 5: 0.970). We used annual survival without hunting to calculate survival during time step 4 (the winter period, which lasts 4.5 months), by $0.9543^{4.5/12}$, which gives a survival during time step 4 of

0.983. Since there was no data on subadult survival during the months after fletching, we also used annual survival of the whole population to calculate subadult survival in time step 3 (0.963), as well as to calculate survival during the last phase of the breeding period, when fledging already took place. The number of eggs (in time step 1) was obtained from one well-studied population (from Hellegatsplaten) during the period 2004-2006 and was 5.03 per breeding pair, which is 2.515 per individual goose. Chick survival (during time step 2) was calculated using all five different colonies considered (van der Jeugd 2013), with data obtained from 2004 till 2007. For all five colonies, data from 2006 and 2007 was available, for four colonies, data from 2005 was available and for two of the five (under which Hellegatsplaten) data from 2004 was available. Chick survival from egg to fledging was on average 0.121. The total survival in time step 2 was calculated as follows: $0.121 \times 0.8346^{1/12} = 0.119$.

Published data about the Baltic population (Larsson and Forslund 1994; van der Jeugd and Larsson 1998) were complemented until 2001 (unpublished data). The full dataset used in our model consisted of data from the colony in Laus Holmar collected between 1984 and 2001. Data on number of eggs was available for all years and the average over the years was used in time step 1 (4.63 per breeding pair, 2.315 per individual parent). Data on chick survival in time step 2 was obtained by taking the survival from egg to fledgling (0.131). Post-fledgling survival (from fledging until arrival on the wintering grounds) was also available (0.911). This was survival over a period of 3 months. During 1 of these months, chicks were still present at the breeding grounds. This period still belonged to time step 2. Therefore, to obtain the complete chick survival in time step 2, the survival until fledging was multiplied with the post-fledging survival to the power of 1/3 ($0.131 \times 0.911^{1/3} = 0.127$). Time step 3, the autumn migration for the subadults, was calculated by using the remaining two months of the post-fledging survival ($0.911^{2/3} = 0.94$). Adult survival was obtained from the same colony. This data is underlying the publication by van der Jeugd et al. (2009) and ranges from 1985 to 2000. Unfortunately, no data was available on distinct survival rates in the different areas. Therefore we used the adult annual survival from the first winter onwards (0.897). The survival per time step was calculated as followed: $0.897^{x/12}$, where x is the number of months which are included in the different time steps (1: 1 month, 2: 2 months, 3: 2.5 months, 4: 4.5 months, 5: 2 months). This led to the following survival rates for the adults in the different time steps (see also Fig. 5): 1: 0.991, 2: 0.982, 3: 0.978, 4: 0.960, 5: 0.982.

Data about time step 1 and 2 of the Russian population originated from (van der Jeugd et al. 2009). Time step 1 (the laying data) was obtained between 2003 and 2005 from a breeding colony in the Kolokolkova Bay in the Barents Sea. Total number of eggs laid per breeding pair was 3.91. This is 1.955 eggs per individual goose. Time step 2 was obtained from the same colony during the same period by multiplying total chick survival (from egg to fledging), which was 0.193, with postfledging survival (from fledging to arrival at the wintering grounds: 0.500) to the power of 1/3, since this period included 3 months, of which one month was part of time step 2 ($0.193 \times 0.500^{1/3} = 0.153$). Time step 3 for the subadults was again calculated by using the remaining two months of the postfledging survival ($0.500^{2/3} = 0.63$). Data on annual survival of adults were obtained from recent years (2003-

2014) from the same colony, using published datasets (van der Jeugd et al. 2009; Lameris 2018). Again, no data was available on survival per area. Therefore, we used annual adult survival from the first winter onwards (0.861). We calculated the survival per time step in the same way we did for the Baltic population, which led to the following survival rates for the adults in the different time steps: 1: 0.988, 2: 0.975, 3: 0.969, 4: 0.945, 5: 0.975.

Calculations population dynamics

All the vital rates calculated above were put into matrices (Fig. 5). These matrices were used to determine the population dynamics (Fig. S1). At the start of the calculations, we assigned a number of individuals to R_0 (100 in this case, but the exact value is of no influence on the outcome of the model). With this number and the matrix of time step 1 (Fig. 5), we could calculate R_1 and J_1. S_1 remained 0, since we did not consider subadults remaining at the wintering grounds. After that, we calculated J_2 and R_2, by multiplying those with the matrix of time step 2. We continued like this until time step 5. The results of time step 5 were then inserted in the first time step, for a calculation of the second year and so on.

We calculated λ, which is a value indicating the growth (when >1) or decline (when <1) of a population from one year to the next. This was calculated by dividing the total number of individuals (the sum over all life stages) in one time step in one year with the total number of individuals in that time step the previous year. All different populations had a λ which was larger than 1, indicating population growth. For the resident population, λ was 1.139, for the Baltic population 1.157 and for the Russian population 1.034.

Sensitivity (= change in λ by a change in the parameter) and elasticity (= relative change in λ by a relative change in a parameter) of λ were calculated for a 1% change in the matrix elements.

References

Lameris TK (2018) Outflying climate change: optimal timing of migratory geese breeding in a warming Arctic. PhD thesis, Universiteit van Amsterdam. , Amsterdam

Larsson K, Forslund P (1994) Population dynamics of the Barnacle Goose (*Branta leucopsis*) in the Baltic area - density-dependent effects on reproduction. J Anim Ecol 63:954-962

van der Jeugd H (2013) Survival and dispersal in a newly-founded temperate Barnacle Goose Branta leucopsis population. Wildfowl 63:72-89

van der Jeugd HP, Eichhorn G, Litvin KE, Stahl J, Larsson K, van der Graaf AJ, Drent RH (2009) Keeping up with early springs: rapid range expansion in an avian herbivore incurs a mismatch between reproductive timing and food supply. Glob Change Biol 15:1057-1071

van der Jeugd HP, Larsson K (1998) Pre-breeding survival of barnacle geese *Branta leucopsis* in relation to fledgling characteristics. J Anim Ecol 67:953-966

Fig. S1 Example life cycle bird: brent goose. The main interactions in the ecosystem contexts are indicated. The arrow indicating the fecundity is both blue and green, since an important carry-over effect has been established in brent geese, in which the ecosystem context of the wintering grounds strongly impacts the reproductive output on the breeding grounds. Interactions in the ecosystem context are indicated with arrows.

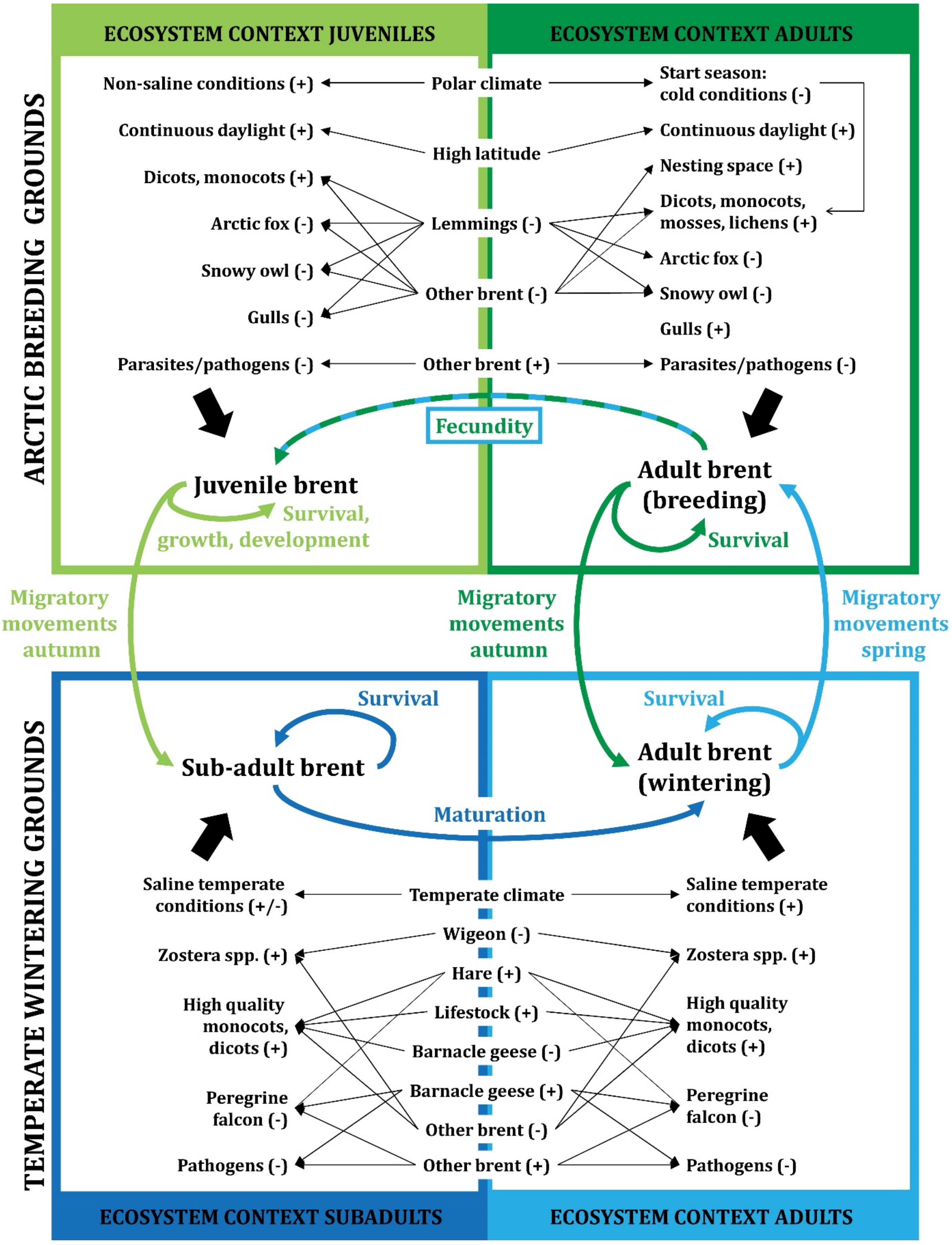

Fig. S2 Example life cycle fish: Pacific salmon (*Oncorhynchus* spp.). Important interactions are indicated. Reproductive adult salmon die after spawing in the freshwater spawning streams. This lack of survival is compensated for by high fecundity. However, survival of reproductive adults salmon, as well as migration back to the oceans are lacking from the diagram.

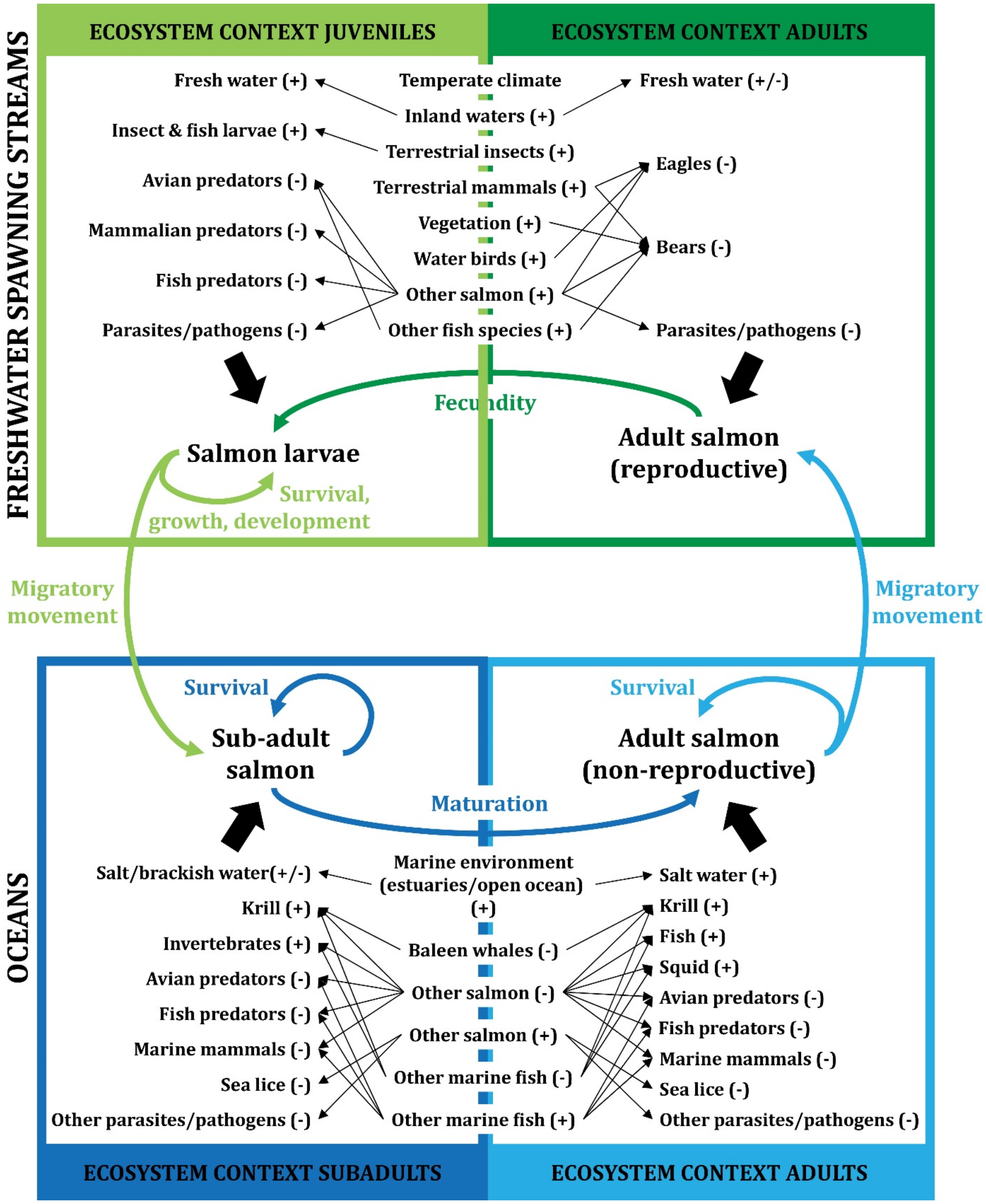

Table S1 Sensitivity (= change in λ by a change in the parameter) and elasticity (= relative change in λ by a relative change in a parameter) of λ for a 1% change in the matrix elements. See Fig. 2 for where the parameters fit in the matrix.

	Resident (Dutch population)			Short-distance migrantion (Swedish population)			Long-distance migration (Russian population)		
Parameter	Nominal value	Sensitivity	Elasticity	Nominal value	Sensitivity	Elasticity	Nominal value	Sensitivity	Elasticity
a_{jr}	2.515	0.109	0.241	2.315	0.113	0.225	1.955	0.089	0.168
a_{rr1}	0.970	0.891	0.759	0.991	0.905	0.775	0.988	0.870	0.832
a_{jj}	0.119	2.309	0.241	0.127	2.051	0.225	0.153	1.135	0.168
a_{rr2}	0.970	0.891	0.759	0.982	0.914	0.775	0.975	0.882	0.832
a_{sj}	0.963	0.285	0.241	0.940	0.277	0.225	0.630	0.276	0.168
a_{nr}	0.963	0.897	0.759	0.978	0.917	0.775	0.969	0.888	0.832
a_{ns}	0.983	0.280	0.241	0.960	0.271	0.225	0.945	0.184	0.168
a_{nn}	0.983	0.879	0.759	0.960	0.935	0.775	0.945	0.910	0.832
a_{rn}	0.970	1.174	1.000	0.982	1.179	1.000	0.975	1.060	1.000
